# Transient MRI changes and neurological deterioration in glioblastoma upon SARS-CoV-2 infection

**DOI:** 10.1007/s00432-024-05963-4

**Published:** 2024-09-28

**Authors:** Thomas Zeyen, Lea L. Friker, Daniel Paech, Niklas Schaefer, Johannes Weller, Valentina Zschernack, Julian P. Layer, Matthias Schneider, Anna-Laura Potthoff, Marit Bernhardt, Christine Sanders, Glen Kristiansen, Michael Hoelzel, Eleni Gkika, Alexander Radbruch, Torsten Pietsch, Ulrich Herrlinger, Christina Schaub

**Affiliations:** 1 Department of Neurooncology, Center for Neurology, University Hospital Bonn, Bonn, Germany; 2 Institute of Neuropathology, University Hospital Bonn, Bonn, Germany; 3 Institute of Experimental Oncology, University Hospital Bonn, Bonn, Germany; 4 Department of Neuroradiology, University Hospital Bonn, Bonn, Germany; 5 Department of Vascular Neurology, Center for Neurology, University Hospital Bonn, Bonn, Germany; 6 Department of Radiation Oncology, University Hospital Bonn, Bonn, Germany; 7 Department of Neurosurgery, University Hospital Bonn, Bonn, Germany; 8 Institute of Pathology, University Hospital Bonn, Bonn, Germany

**Keywords:** Glioblastoma, SARS-CoV-2, COVID-19, MRI, Pseudoprogression, Tumor microenvironment

## Abstract

**Purpose:**

Little is known about the effect of SARS-CoV-2 infection on glioblastoma (GBM) growth, metabolism, and prognosis. Immunological changes within GBM tissue are potentially symptomatic, underlining the urgent need for a better understanding of this phenomenon. To date, the complex underlying biology has not been fully elucidated. A decisive role of the tumor microenvironment (TME) and the components of the immune system acting within it is assumed.

**Methods:**

Immunohistochemical staining of SARS-CoV-2 spike protein and immune cell infiltration of TME was performed on the tumor tissue of one patient. This patient developed hemiparesis 14 days after symptomatic SARS-CoV-2 infection, leading to tumor diagnosis. Subsequently and after biopsy, there was an unexpectedly good response to chemotherapy only. In looking for further evidence of the potential of SARS-CoV-2 to influence the course of GBM, two additional adult patients that had transient MRI changes and neurological deterioration following SARS-CoV-2 infection were evaluated.

**Results:**

In the patient for whom neurological deterioration in the course of SARS-CoV-2 led to GBM diagnosis, immunohistochemistry revealed virus-specific protein accumulation in the tumor cells, microglial activation, and the formation of T-cell nodules. In the other two patients, the findings were compatible with symptomatic pseudoprogression that occurred in a temporal relationship with SARS-CoV-2 infection.

**Conclusion:**

The results indicate a possible association between clinically relevant changes in GBM biology and SARS-CoV-2 infection, with histological confirmation of SARS-CoV-2-associated changes within the tumor tissue. The exact pathomechanism and underlying inflammatory pathways require further investigation.

## Introduction

Pseudoprogression occurs in up to 50% of patients with glioblastoma (GBM) (Wick et al. 2016; Ellingson et al. 2017) and is clinically relevant for therapy-guiding decisions (Young et al. 2023; Galldiks et al. 2017). It can be defined as progressive MRI changes (e.g., enlarged or new tumor lesions) that remain stable or regress without further therapy. Histopathological studies describe dominant features such as bland necrosis, reactive gliosis, edema, demyelination, vascular hyalinization, and influx of immune cells (Melguizo-Gavilanes et al. 2015; Le Fèvre et al. 2021). Most pseudoprogressions occur within the first three months of radiation therapy (RT) (Ellingson et al. 2017). Immunological and inflammatory processes that affect GBM biology are diverse and far from being understood. Viral particles may play a relevant role, as cytomegalovirus (CMV) reactivation after RT is one of the most common virus-associated alterations causing encephalopathy in patients with GBM (Goerig et al. 2016). Some experimental therapeutic concepts such as local oncolytic virus therapy (Todo et al. 2022; Desjardins et al. 2018; Gállego Pérez-Larraya et al. 2022; Friedman et al. 2021; Lang et al. 2018; Ling et al. 2023) even rely on inducing intratumoral inflammation causing enlargement of the targeted lesion before RT and is referred to as “immunoprogression” (Todo et al. 2022). Whether infection at the tumor site is generally associated with prolonged survival remains a matter of debate. While reports of oncolytic virus trials find indications for survival-prolonging effects (Ling et al. 2023), and such an effect is also discussed for early postoperative wound infections after GBM resection (Solár et al. 2022; Chen et al. 2017; Hounchonou et al. 2024), reactivation of CMV upon RT (+ dexamethasone) for GBM tends to be associated with inferior survival (Goerig et al. 2016; Yang et al. 2022). For local intercurrent viral central nervous system (CNS) infections, including SARS-CoV-2, in GBM patients, no reports are available. Of note, a recent article published by Gregory et al. (2024) elaborated that GBM patients may have accelerated disease progression in the first two months after SARS-CoV-2 infection. They highlight the need to further investigate the tumor microenvironment (TME) changes that could be associated with this observation. On the contrary, there is one multi-institutional clinical report without histological analyses showing no impact of systemic SARS-CoV-2 infection on the survival of patients with GBM (Vogel et al. 2023). For the first time, we report here three GBM patients that had clinically relevant changes in MRI following SARS-CoV-2 infection consistent with pseudoprogression in two cases and one patient with atypically good therapy response to chemotherapy only and detection of viral GBM cell infection as well as associated TME changes.

## Materials and methods

### Patients

In 2022, patient 1 of this series presented to our Neurooncology center with a brain MRI showing a new T1-enhancing lesion in the left basal ganglia, suspicious for GBM. He developed hemiparesis shortly after SARS-CoV-2 infection. The diagnosis of GBM Isocitratedehydrogenase (IDH)-wildtype (CNS WHO grade 4) was confirmed by tissue analysis after stereotactic biopsy. Interestingly, he had an unexpected favourable clinical and radiological response after three courses of alkylating chemotherapy with temozolomide (TMZ) only. We wondered whether the temporal relationship with SARS-CoV-2 infection was a coincidence or whether there could be a closer connection behind it. In looking for further evidence to this, records of the Neurooncology Center at the University Hospital Bonn were screened for patients that were treated within 2022 and that fulfilled the following criteria: (1) Diagnosis of IDH-wildtype GBM (according to WHO 2021). (2) Acute neurological deterioration upon SARS-CoV-2 infection (detected via PCR-analysis). (3) MRI showing progressive or new T1-enhancement and fluid-attenuated inversion recovery (FLAIR)/T2 lesions that were regressive in subsequent scans. Neurological deterioration was assessed according to the Neurology Assessment in Neuro-Oncology (NANO) scale (Nayak et al. 2017). The Karnofsky Performance Scale (KPS) was used to assess the patient’s general fitness (Friendlander & Ettinger 2009). Progression assessment on 3T MRI was performed according to the response assessment in the neuro-oncology (RANO) criteria (Youssef & Wen 2024). MRI analyses also included MRI perfusion (fast field echo - echo-planar imaging (FFE EPI) perfusion) as an imaging modality that has the potential to distinguish progressive GBM changes from pseudoprogressive (and potentially immune-related) changes (Zhang et al. 2022).

### Tumor tissue samples and immunohistochemistry

Formalin-fixed paraffin-embedded tissue (FFPE) samples were cut into 4 μm thick slices and subsequently processed for standard H&E staining (Mayer’s hemalum; 2E-038 Waldeck GmbH & Co. KG, Münster and eosin Y (1%); 1159350, Merck, Darmstadt, Germany). Immunohistochemistry was performed on a Ventana Benchmark XT Immunostainer (Roche Ventana, Darmstadt, Germany). Therefore, the following primary antibodies were used: mouse anti-MAP2 (clone HM-2, Sigma-Aldrich, St. Louis, MO, USA, dilution 1:20000), mouse anti-Ki-67/MIB1 (clone Ki-67P, Dianova, Hamburg, Germany, dilution 1:1000), mouse anti-CD68 (cluster of differentiation; clone KP1, M 0814, Agilent Technologies, Santa Clara, CA, USA, dilution 1:1000), mouse anti-CD3 (Novocastra™, Leica Biosystems, Newcastle, England dilution 1:50), mouse anti-CD8 (clone C8/144B, Agilent Technologies, dilution 1:100) and SARS-CoV-2 spike protein (clone 1A9, GTX632604, GeneTex Inc., Irvine, CA, USA, dilution 1:50).

## Results

### Patients

Three adult patients (aged 60–80 years) with diagnosis of GBM were analyzed. The vaccination status in patients 1 and 2 was unknown, patient 3 had two vaccinations prior to SARS-CoV-2 infection. Table 1 provides information on the survival and clinical data of the patient cohort. In the time interval of 10–14 days after symptom onset of COVID-19 with moderate general symptoms, the patients experienced significant neurological deterioration. Duration of SARS-CoV-2 infection (defined as time from first positive PCR-result to first negative PCR-result) was 12 days in patient 1 and 3, and 25 days in patient 2. Patient 1 had a NANO score of four points and patients 2 and 3 had an increase in the NANO score of five points, respectively.

**Table 1:** Survival and clinical data. Summary of clinical patient parameters

	patient 1	patient 2	patient 3
*MGMT* methylation status	methylated	non-methylated	non-methylated
KPS at SARS-CoV-2 infection	80%	60%	80%
NANO score (increase in points)	4 (n.a.)	9 (+ 5)	7 (+ 5)
first PFS (RANO)	7 months	8 months	8 months
OS	9 months	11 months	12 months

MGMT = O-6-methylguanine-DNA methyltransferase, KPS = Karnofsky Perfomance score; SARS-CoV-2 = severe acute respiratory syndrome coronavirus 2, NANO = neurologic assessment in neuro-oncology; PFS = progression free survival; OS = overall survival; n.a. = not applicable)

**Fig. 1:**
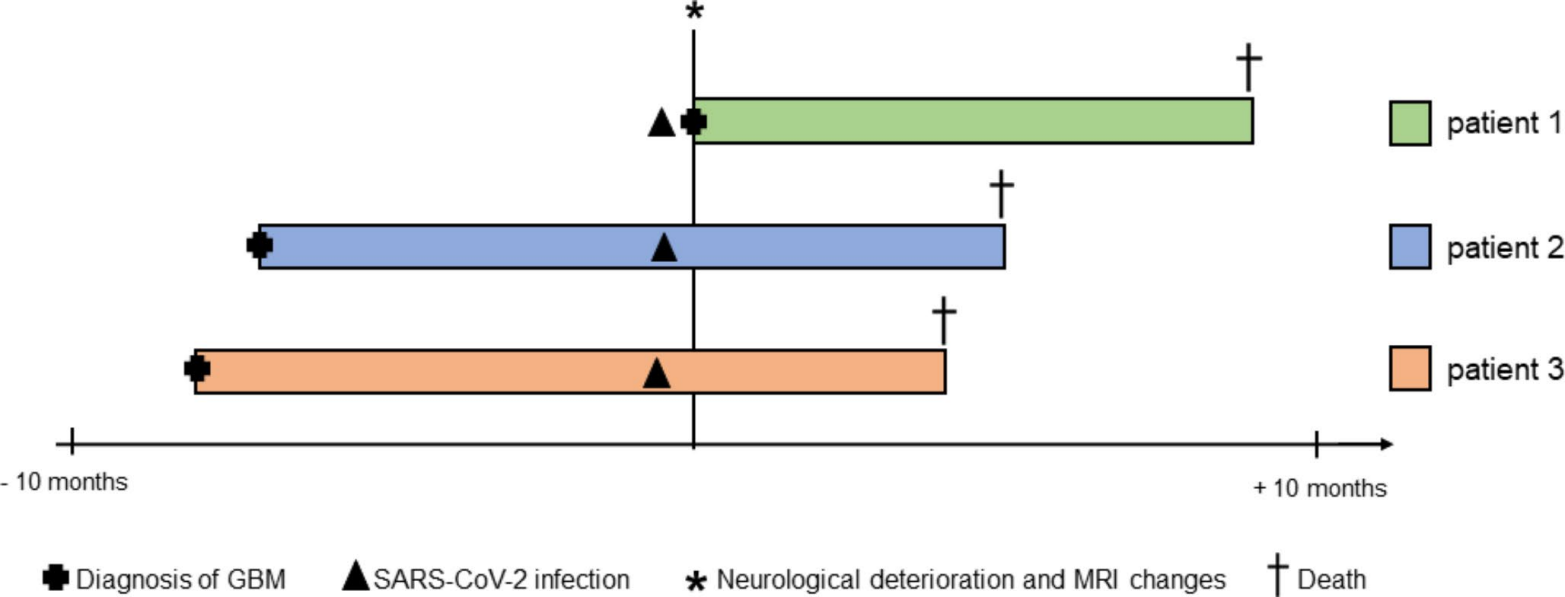
Swimmers plot. Provides a swimmers plot showing the time line for each patient from diagnosis of GBM to death and illustrating the time point of SARS-CoV-2 infection with subsequent neurological deterioration

SARS-CoV-2 infection with subsequent neurological deterioration occurred at the following stages of their brain tumor disease: In patient 1, the development of neurological symptoms in the context of SARS-CoV-2 led to the detection of a brain tumor that was subsequently diagnosed as GBM upon stereotactic biopsy. Patient 2 previously underwent gross total resection, RT with concomitant TMZ followed by four courses of adjuvant TMZ therapy (up to 200 mg/m2/day), and patient 3 underwent gross total resection followed by RT and three courses of combined CCNU/TMZ therapy (Herrlinger et al. 2019). The decision for combined treatment with CCNU/TMZ in patient 3, albeit a non-methylated MGMT promotor, was made on an individual basis, and treatment was started in an external center. Figure 1 provides an illustration of each patients disease course related to SARS-CoV-2 infection with subsequent neurological deterioration and MRI changes.

### MRI

**Fig. 2:**
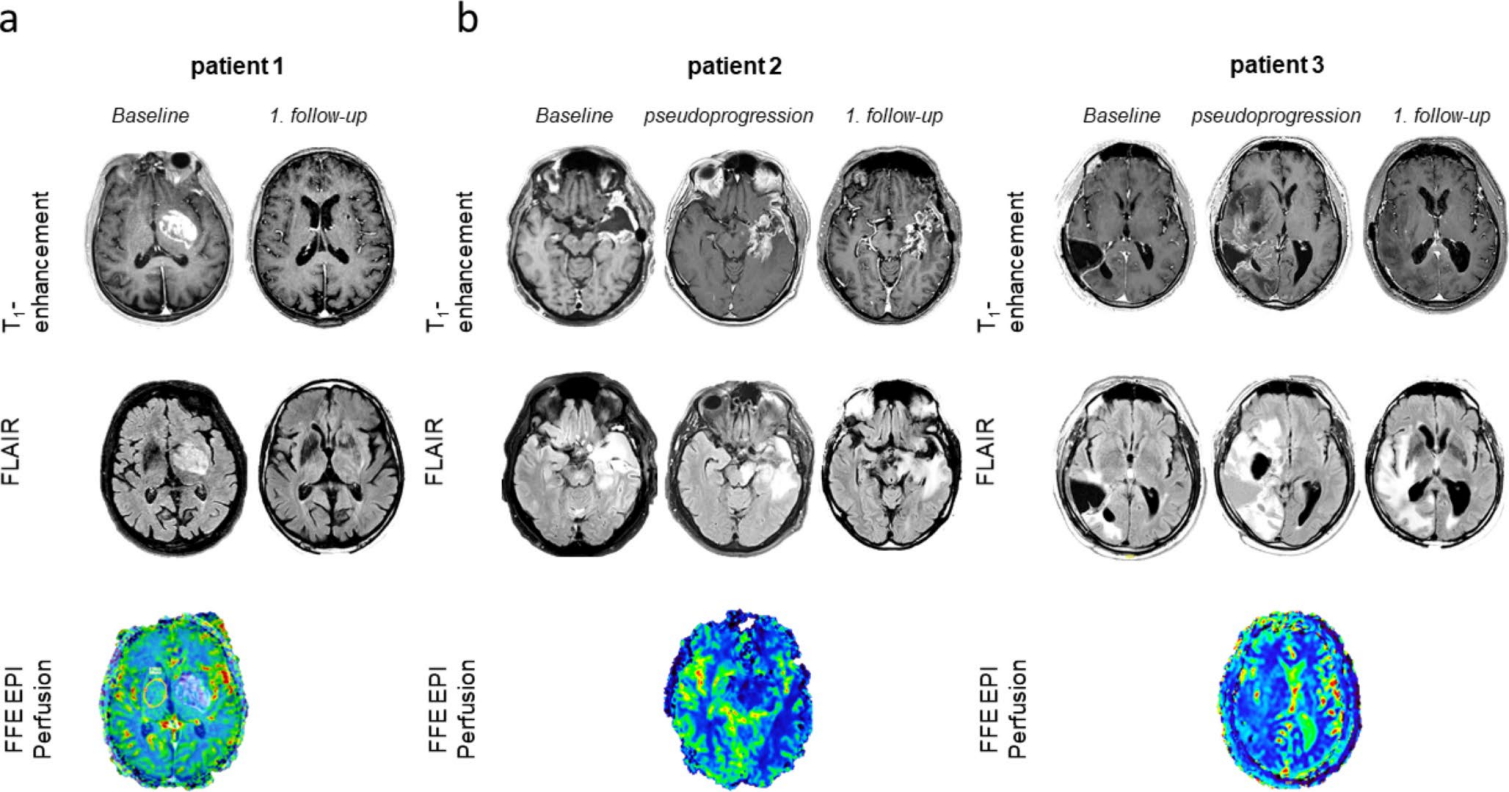
MRI findings. **(a)** shows MRI findings in patient 1 including the T1-enhancement and FLAIR studies. “Baseline” shows first MRI of diagnosis, “1. follow-up” shows the first MRI after TMZ therapy and demonstrates regressive findings MRI before increase in T1-anhancement / FLAIR. **(b)** shows MRI findings in patients 2 and 3 including the T1-enhancement and FLAIR studies. “Baseline” shows MRI with increased T1-enhancement/FLAIR hyperintensities, “Pseudoprogression” shows the MRI with corresponding increase. “1. Follow-up” shows the first MRI after observation of the increase and demonstrates regressive findings´. In **(a) and (b)** the bottom row shows FFE EPI perfusion imaging is shown indicating no hyperperfusion in the respective T1-enhancement area

In patient 1, MRI that led to the diagnosis of GBM showed marked T1 contrast enhancement in the left-sided basal ganglia (Fig. 2a). After diagnosis primary therapy with three standard courses of TMZ monotherapy at 150 mg/m² body surface was initiated. Radiotherapy was omitted due to age in conjunction with a methylated MGMT promotor in this tumor. The first follow-up MRI showed an unexpected and remarkable reduction in residual contrast enhancement and regressive FLAIR hyperintensities. MRI perfusion imaging of the first brain MRI unexpectedly showed no signs of hyperperfusion in the T1-enhancement area, which is regularly seen in pseudoprogressive changes, radionecrosis, or immunological changes. Patients 2 and 3 presented with worsening of neurological symptoms and an increase in contrast-enhancing T1 lesions and FLAIR hyperintensities during adjuvant TMZ therapy that regressed in the first follow-up MRI six weeks later without a change in antitumoral therapy (Fig. 2b). Again, no relevant hyperperfusion was observed in the MRI that showed an increase in contrast-enhancing T1 lesions and FLAIR hyperintensities. Both patients received dexamethasone therapy afterwards (highest dose, 12 mg and 4 mg, respectively).

### Histology

**Fig. 3:**
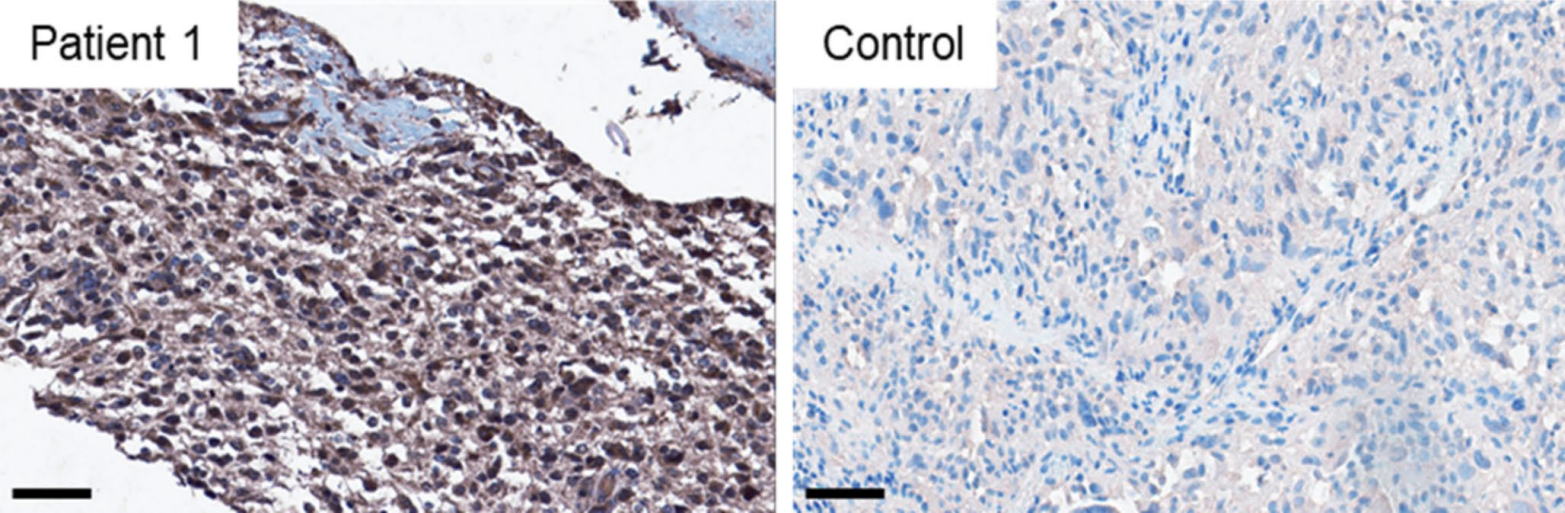
Immunohistochemical staining of SARS-CoV-2 spike protein. Immunohistochemical staining with antibodies against SARS-CoV-2 spike protein. Patient 1 (left) shows a severe accumulation of spike protein within the tumor tissue, whereas the tumor of the control patient (right) reveals a negative staining, implying the absence of spike protein. Scale bars equate 60 μm. SARS-CoV-2 = severe acute respiratory syndrome coronavirus 2

**Fig. 4:**
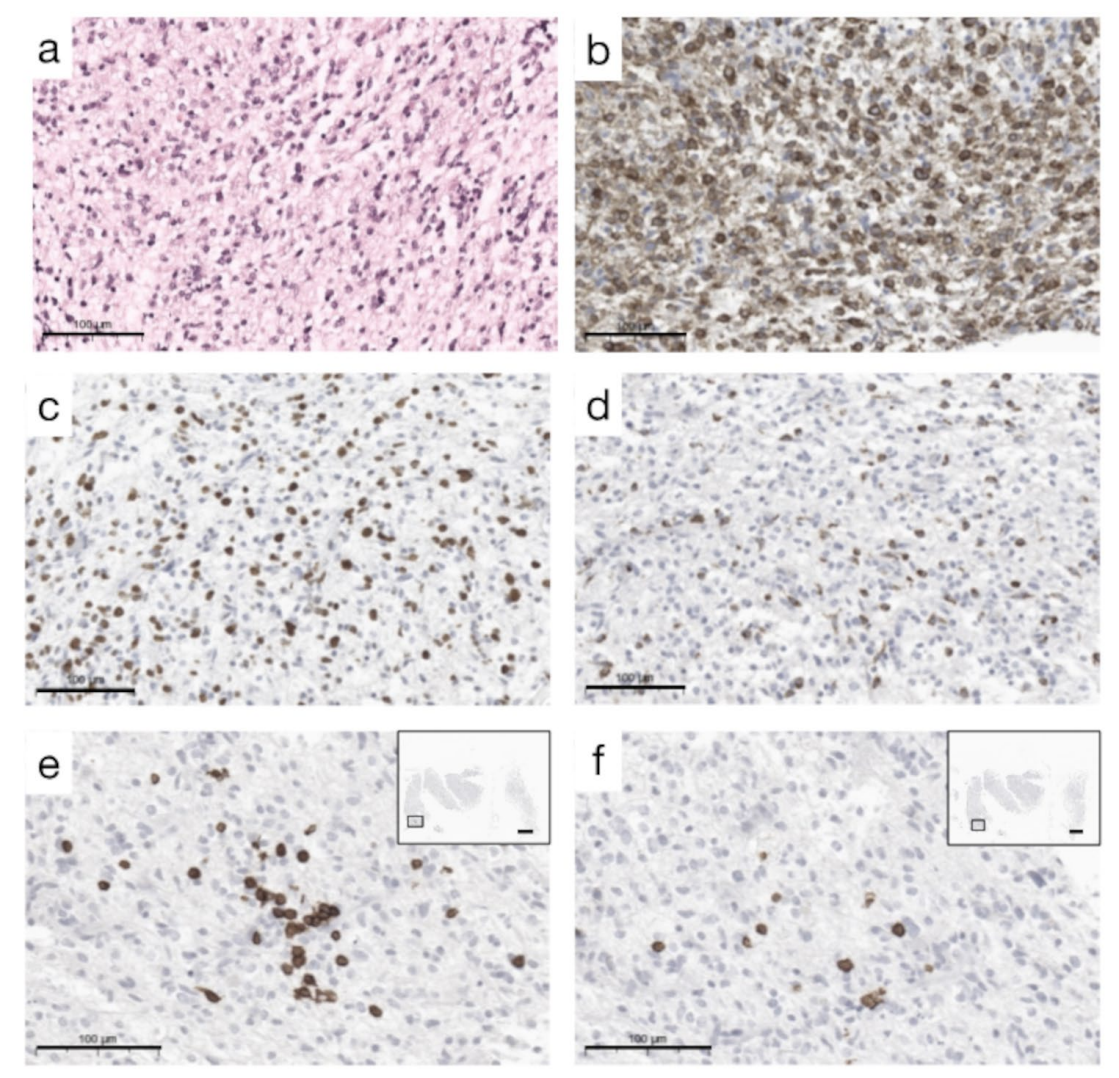
Histological and immunohistochemical analysis of TME. Histological and immunohistochemical analysis of immune infiltration in GBM tumor tissue. **(a)** H&E, **(b)** Map2c, **(c)** Ki-76, **(d)** CD68, **(e**,** f)** Focal T-cell aggregation **(e)** CD3, **(f)** CD8, showing the same tumor area. Scale bar equates 100 μm and 500 μm in overview images, upper right **(e**,** f)**. GBM = glioblastoma (CNS WHO grade 4) CD = cluster of differentiation

In patient 1, tumor tissue taken 16 days after COVID-19 onset and one day after the first MRI provided the opportunity to analyze it for signs of local SARS-CoV-2 infection and immune cell infiltration. Immunohistochemical staining with antibodies against SARS-CoV-2 spike protein showed an accumulation of spike protein in GBM tumor cells and macrophages (Fig. 3), which was not seen in a control glioma patient with tumor resection in 2016 prior to the COVID-19 pandemic. Activation of CD68-positive microglia (Fig. 4d) and the formation of so-called immune cell “nodules” containing CD3- and CD8-positive T-cells in the tumor tissue could be demonstrated (Fig. 4e and f). With antibodies against CD20, only single B cells were detected (not shown).

### Survival

Patients 1 and 2 did not receive second-line therapy, and patient 3 received CCNU for second-line therapy. Of note, patient 3 also received two courses of bevacizumab (7.5 mg/kg bodyweight) for symptomatic treatment of therapy-associated changes due to ineffective steroid therapy. Bevacizumab was applied after the follow-up MRI (as presented in Fig. 2b). In all three patients, overall survival (OS) was substantially shorter than the median for their age and MGMT promotor methylation group (patient 1: OS 9 months vs. an age- and MGMT-adjusted median OS of 18 months (Perry et al. 2017); patients 2 and 3: OS 11 months and 12 months, respectively, compared to an age- and MGMT-adjusted median OS of 17 months (Chinot et al. 2014).

## Discussion

This study reveals clinically and radiologically relevant changes in patients with GBM upon SARS-CoV-2 infection. The onset of neurological deterioration within 10–14 days of the onset of systemic SARS-CoV-2 infection implies a temporal relationship. Immunohistochemical analyses available from biopsy specimens of one of the patients confirmed accumulation of spike protein in the tumor cells and a SARS-CoV-2 associated TME reaction. These links have not been described previously.

COVID-19-associated brain tissue changes have been reported radiologically and neuropathologically. Severe SARS-CoV-2 infection may cause neocortical brain degeneration (Bendella et al. 2023). The neuropathological findings reported here with increased microglia activation and formation of immune cell “nodules” are in line with postmortem findings in the CNS of patients with lethal SARS-CoV-2 infection (Schwabenland et al. 2021). Our observation of SARS-CoV-2 spike protein accumulation in GBM tumor cells has not been previously described. This finding, however, is not surprising considering the fact that angiotensin-converting enzyme-2 (ACE2), transmembrane serine protease-2 (TMPRSS2), and particularly neuropilin-1 (NRP1), all facilitating the entry of SARS-CoV-2 into the CNS, are also found in GBM tissue, as well as in organoids and cell lines (Suarez-Meade et al. 2023). NRP1 gene expression is upregulated in GBM compared to healthy brain tissue (Suarez-Meade et al. 2023). The short overall survival times in our three patients speak against a positive influence of SARS-CoV-2 infection on the prognosis of GBM.

From a clinical perspective, this is in line with the work of Gregory et al., where the authors described that GBM patients may have accelerated disease progression in the first two months after SARS-CoV-2 infection (Gregory et al. 2024). From a translational perspective, SARS-CoV-2 infection combined with higher expression of NRP1 in GBM tissue has the potential to promote the infiltration of TME with immunosuppressive immune cells (Suarez-Meade et al. 2023). This may have contributed to the worsened prognosis, as an immunosuppressive TME is expected to be a major reason for therapy resistance in gliomas (Lin et al. 2024). The decisive role of the immunosuppressive TME in therapy resistance has also been addressed in new therapeutic approaches. For instance, the GLIOSTAR trial aims to investigate the potential effect of adding the pro-inflammatory cytokine tumor necrosis factor (TNF) combined with a tumor-targeted antibody (L19) to the standard recurrence therapy with lomustine (Look et al. 2023; NCT03779230). The targeting of the TME rather than the induction of necrosis sets SARS-CoV-2 apart from oncolytic viruses such as herpes viruses aiming at GBM cell necrosis, subsequent inflammation, and survival prolonging effects.

Interestingly, all reported patients were infected with SARS-CoV-2 by 2022. Indeed, SARS-CoV-2 variants have been shown to differ in their neuropathogenicity (Bauer & Riel 2023). For example, the D614G variant appears to be more neurotropic and neurovirulent than the Delta and Omicron variants. The neuroinflammation observed with D614G might be associated with an increased ability to replicate in the olfactory mucosa and invade the CNS (Bauer et al. 2022). Moreover, it is likely that in the early phase of the pandemic, patients suffering from chronic oncological diseases were carefully protected from SARS-CoV-2 infection. The availability of vaccines and medications enabled them to reparticipate in social activities. In conclusion, many factors such as self-protection, social isolation, virulence, neuropathogenicity of the virus variant, and vaccination status may influence the time point when potentially SARS-CoV-2-associated GBM changes could be observed.

Our study has several limitations. As the observation was made in only three patients (immunohistochemistry of SARS-CoV-2 infected tumor tissue in only one patient) that do not form a representative cohort, conclusions cannot claim proven causality regarding SARS-CoV-2 infections and changes in the tumor that are compatible with pseudoprogression. In addition, the vaccination status of two patients and information on the exact virus strain of all patients was unfortunately unavailable. Apart from that, quantitative data on virus load or cytokine levels in blood or cerebrospinal fluid (CSF) were not available.

In summary, the present report suggests a previously unknown interaction between SARS-CoV-2 infection and the GBM microenvironment that may ultimately lead to the clinical and radiological phenomenon of pseudoprogression. Further research is needed to determine the exact pathomechanism and frequency of SARS-CoV-2-associated pseudoprogression in post-pandemic days.

## Data Availability

Data will be provided by Thomas Zeyen (first author) upon reasonable request.
